# CCR8 marks highly suppressive Treg cells within tumours but is dispensable for their accumulation and suppressive function

**DOI:** 10.1111/imm.13337

**Published:** 2021-05-09

**Authors:** Sarah K. Whiteside, Francis M. Grant, David S. Gyori, Alberto G. Conti, Charlotte J. Imianowski, Paula Kuo, Rabab Nasrallah, Firas Sadiyah, Sergio A. Lira, Frank Tacke, Robert L. Eil, Oliver T. Burton, James Dooley, Adrian Liston, Klaus Okkenhaug, Jie Yang, Rahul Roychoudhuri

**Affiliations:** ^1^ Department of Pathology University of Cambridge Cambridge UK; ^2^ Immunology Programme Babraham Research Campus Babraham Institute Cambridge UK; ^3^ Department of Physiology Semmelweis University Budapest Hungary; ^4^ Mount Sinai School of Medicine Immunology Institute New York NY USA; ^5^ Department of Hepatology & Gastroenterology Campus Virchow‐Klinikum (CVK) and Campus Charité Mitte (CCM) Charité Universitätsmedizin Berlin Berlin Germany; ^6^ Department of Surgery Memorial Sloan Kettering Cancer Center New York NY USA

**Keywords:** Treg, CCR8, Foxp3, CD4^+^ T cells, CD8^+^ T cells, cancer, immunotherapy

## Abstract

CD4^+^ regulatory T (Treg) cells, dependent upon the transcription factor Foxp3, contribute to tumour immunosuppression but are also required for immune homeostasis. There is interest in developing therapies that selectively target the immunosuppressive function of Treg cells within tumours without disrupting their systemic anti‐inflammatory function. High levels of expression of chemokine (C‐C motif) receptor 8 (CCR8) discriminate Treg cells within tumours from those found in systemic lymphoid tissues. It has recently been proposed that disruption of CCR8 function using blocking anti‐CCR8 antibodies results in reduced accumulation of Treg cells within tumours and disruption of their immunosuppressive function. Here, using *Ccr8* ^−/−^ mice, we show that CCR8 function is not required for Treg cell accumulation or immunosuppression in the context of syngeneic MC38 colorectal adenocarcinoma and B16 melanoma tumours. We observed high levels of CCR8 expression on tumour‐infiltrating Treg cells which were abolished in *Ccr8* ^−/−^ mice. High levels of CCR8 marked cells with high levels of suppressive function. However, whereas systemic ablation of Treg cells resulted in strikingly diminished tumour burden, growth of subcutaneously implanted tumours was unaffected by systemic CCR8 loss. Consistently, we observed minimal impact of systemic CCR8 ablation on the frequency, phenotype and function of tumour‐infiltrating Treg cells and conventional T (Tconv) function. These findings suggest that CCR8 is not required for Treg cell accumulation and immunosuppressive function within tumours and that depletion of CCR8^+^ Treg cells rather than blockade of CCR8 function is a more promising avenue for selective immunotherapy.

## Graphical abstract

There is interest in developing therapies that selectively target the immunosuppressive function of Treg cells within tumours without disrupting their systemic anti‐inflammatory function. It has recently been proposed that disruption of CCR8 function using blocking anti‐CCR8 antibodies results in reduced accumulation of Treg cells within tumours and disruption of their immunosuppressive function.​We show that CCR8 marks highly suppressive Treg cells within tumours but is not required for Treg cell accumulation and immunosuppressive function within tumours, suggesting that depletion of CCR8^+^ Treg cells rather than blockade of CCR8 function is a more promising avenue for selective immunotherapy.

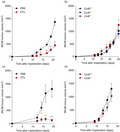

**Abbreviations**
ADCC: antibody‐dependent cellular cytotoxicity
APCs: antigen‐presenting cells
*Areg*
: amphiregulin
C‐C motif: chemokine
CCR8: receptor 8
CTV: CellTrace Violet™
DTR: diphtheria toxin receptor
DTx: diphtheria toxin
EGFP: enhanced green fluorescent protein
NK: natural killer
(PPAR)‐γ: peroxisome proliferator‐activated receptor
Tconv: conventional T
Th: T helper
Treg: regulatory T
WT: wild‐type

## INTRODUCTION

Tumours grow in immunocompetent hosts despite the ability of cells of the adaptive immune system to recognise and kill cancer cells. In part, this phenomenon is attributable to the process of immunosuppression. Tumour immunosuppression is dependent upon a number of peripheral tolerance mechanisms normally employed to prevent unwanted inflammation and autoimmune responses. Therapeutic approaches aimed at disrupting tumour immunosuppression would ideally do so without disturbing systemic peripheral tolerance. CD4^+^ regulatory T (Treg) cells are a suppressive T‐cell subset required to prevent autoimmune and allergic inflammation [1, 2, 3]. Treg cells are often found at high relative frequencies within tumours where they limit immune‐mediated rejection of disease [4, 5, 6]. Consistent with their suppressive function, low Treg to conventional T (Tconv) cell ratios are associated with favourable survival in several types of cancer including ovarian cancer [7, 8], breast cancer [9], non‐small‐cell lung cancer [10], hepatocellular carcinoma [11], renal cell cancer [12], pancreatic cancer [13], gastric cancer [14], cervical cancer [15] and colorectal cancer [16]. Thus, Treg cells diminish both autoimmune and allergic inflammation, but also hinder effective immune responses against tumours. There is a need to develop therapies that selectively target the immunosuppressive function of Treg cells within tumours without disrupting their systemic anti‐inflammatory function.

Recent evidence suggests that Treg cells within different tissues exhibit distinct molecular profiles and functional characteristics. For instance, adipose tissue Treg cells, which express the transcription factor peroxisome proliferator‐activated receptor (PPAR)‐γ, are critical regulators of tissue metabolism and insulin sensitivity [17, 18]. Treg cells in skeletal muscle expressing IL‐33 contribute to muscle repair through expression of molecules such as the epidermal growth factor receptor ligand amphiregulin (AREG) [19, 20]. Recent studies have now shown that Treg cells infiltrating a variety of human tumours, including breast, colorectal and non‐small‐cell lung cancers, exhibit an altered transcriptional profile [21, 22]. These studies showed that Treg cells within tumours are highly activated when compared to systemic and/or normal tissue Treg cells. Among the identified differences was highly increased expression of the chemokine (C‐C motif) receptor 8 (*Ccr8*) gene, encoding CCR8, within tumour‐infiltrating Treg cells as compared with Treg cells found in other tissues.

Chemokines are small (∼8–14 kDa) secreted proteins, structurally similar to cytokines, which regulate cell signalling and trafficking through interactions with a subset of seven transmembrane G protein‐coupled receptors called chemokine receptors [23]. The chemokine receptor CCR8 is a receptor for CCL1 (in humans and mice) and CCL18 (in humans) [24, 25]. CCR8 is expressed on Treg cells, a subset of type helper (Th)‐2 cells, monocytic cells and natural killer (NK) cells [24, 26, 27, 28, 29]. CCR8 has been proposed to play a role in allergic inflammation through loss‐of‐function studies in mice [28], although the extent of its involvement is unclear, with contradictory results in the literature [30, 31]. CCR8 signalling is thought to contribute to Treg cell suppressive function and has been found to promote donor Treg cell survival in a murine model of graft‐versus‐host disease [32]. Moreover, CCR8 signalling by CCL1 has been proposed to potentiate Treg cell proliferation and suppressive function in the context of inflammation of the central nervous system [33].

A recent study has suggested that CCR8 function is required for Treg cell‐mediated tumour immunosuppression [34]. Antibodies with proposed blocking activity were shown to reduce Treg cell accumulation within tumours, drive Tconv cell activation and reduce tumour growth. In addition to blockade, antibodies can induce cell depletion through antibody‐dependent cellular cytotoxicity (ADCC) and fixation of complement via the classical pathway of complement activation. Indeed, surface plasmon resonance experiments have shown that rat IgG2b antibodies of different specificity bind all mouse fixed chain receptors with relatively high affinity, such that their biological effect was reduced in *Fcer1g*‐deficient mice [35]. These findings raise the untested possibility that the IgG2b antibody used in prior experiments to determine the function of CCR8 in tumour immunity depleted Treg cells via ADCC in addition to blocking CCR8 function [34]. Therefore, the function of CCR8 in tumour immunity remains unclear.

In this study, we examined the function of CCR8 in tumour immunosuppression using mice in which CCR8 expression has been genetically ablated. We confirmed high levels of CCR8 expression on tumour‐infiltrating Treg cells, which was abolished on cells from *Ccr8* ^−/−^ mice. Whereas systemic ablation of Treg cells resulted in strikingly diminished tumour growth, growth of subcutaneously implanted tumours was unaffected by systemic CCR8 loss. Consistently, we observed minimal impact of systemic CCR8 ablation on the frequency, phenotype and function of tumour‐infiltrating Treg cells. These findings suggest that CCR8 is not required for Treg cell accumulation and immunosuppressive function within tumours and that depletion of CCR8^+^ Treg cells rather than blockade of CCR8 function may provide a means of selective immunotherapy.

## METHODS

### Mice

*Foxp3* ^EGFP‐DTR^ mice, originally described by Kim et al [36], were obtained from Jackson Laboratories. *Ccr8* ^−/−^ mice were a kind gift from Sergio Lira [26] and Frank Tacke. Animals were genotyped using a custom genotyping service provided by Transnetyx^®^ Inc. All mice were housed at the Babraham Institute Biological Services Unit or the Cambridge University Biomedical Services Gurdon Institute animal facilities. Experiments were performed using mice 8–14 weeks of age, with male and female mice equally distributed into experiment and control groups. Tumour measurements were completed by an independent investigator who was not aware of treatment groups or genotypes. Experiments were repeated 2–4 times using 3–8 mice per group. All animal experiments were conducted in accordance with UK Home Office guidelines and were approved by the Babraham Institute and/or University of Cambridge Animal Welfare and Ethics Review Board.

### Depletion of Treg cells with diphtheria toxin

Diphtheria toxin (DTx) from *Corynebacterium* *diphtheriae* (Sigma‐Aldrich) was obtained in lyophilized powder form and reconstituted in sterile double‐distilled water according to the manufacturer's instructions. Solutions for injection were made up in sterile PBS to a dose of 25 μg/kg. To achieve transient depletion of Treg cells in *Foxp3* ^EGFP‐DTR^ mice, DTx was administered via intraperitoneal injection in 100 μl on days 7, 9, 11 and 14 after tumour implantation.

### MC38 and B16‐F10 heterotopic subcutaneous tumour implantation model

MC38 colon carcinoma cells were purchased from Kerafast. B16‐F10 melanoma cells were purchased from ATCC. Cell lines were passaged in DMEM (Invitrogen) supplemented with 10% FCS and antibiotics. 3·5 × 10^5^ − 2 × 10^6^ MC38 cells in 100 µl PBS or 1·25 × 10^5^ B16‐F10 cells in 100 µl PBS were injected subcutaneously into the right flanks of mice, and tumours were measured with digital callipers at serial time‐points after implantation as previously described [37].

### Suppression of Tconv cells by Tregs

The suppressive capacity of Treg cells was tested as previously described [38]. CCR8^+^ and CCR8^−^ Treg cells were FACS sorted from MC38 tumours of *Foxp3* ^EGFP^^‐^^DTR^ mice. Naïve CD4^+^ Tconv cells (CD25^−^ CD44^−^ CD62L^+^) were obtained from the spleens of WT CD45.1 mice via florescence‐activated cell sorting (FACS) and stained with CellTrace Violet™ (CTV) according to the manufacturer's protocol (Thermo Fisher Scientific). Treg cells and Tconv cells were plated in a 1:4 ratio in the presence of anti‐CD3 (BioLegend 1 µg/ml) and *Rag2* ^−/−^ antigen‐presenting cells (APCs). Naïve Tconv cells cultured without Treg cells were used as the proliferating control. Cell division was evaluated after 4 days of culture.

### Flow cytometry analysis

Tumour samples were digested using collagenase and DNase for 30 min at 37°C, and Lympholyte^®^ (Cedarlane) was used to isolate lymphocytes from tumours. Cell suspensions were filtered using 40µm cell strainers (BD Biosciences). Spleens were mechanically dissociated over a 40µm cell strainer. Red blood cells were lysed using ACK Lysing Buffer (Gibco). Cells were stained with the Fixable Viability Dye eFluor™ 780 (Thermo Fisher Scientific) to discriminate between live and dead cells and then incubated with the following surface antibodies for 30 min on ice: anti‐TCRβ PE (H57‐597), anti‐CD8 PE‐Cy7 (53–6·7), anti‐CD25 APC (PC61.5), anti‐CD44 PerCP‐Cyanine5.5 (IM7), anti‐CD45.1 APC (A20) from eBioscience; anti‐CD4 BUV395 (GK1.5), anti‐CD62L BUV737 (MEL‐14) from BD Biosciences and anti‐CCR8 BV421 (SA214G2), anti‐Thy1.2 BV605 (53‐2.1) from BioLegend. Cells were stimulated with phorbol 12‐myristate 13‐acetate (PMA) and ionomycin and blocked with brefeldin A (BFA) for 4 h in RPMI 1640 complete medium. Intracellular antibodies anti‐Foxp3 APC (FJK‐16S), anti‐IFN‐γ FITC (XMG1.2) and anti‐TNF PE‐Cy7 (MP6‐XT22) were purchased from eBioscience and used with the eBioscience Foxp3/Transcription Factor Staining Buffer Set (Invitrogen, Thermo Fisher Scientific) according to the manufacturer's protocol. Samples were analysed using BD Fortessa and Beckman Coulter CytoFLEX analysers. After analysis, data were analysed using FlowJo software (Tree Star, Inc.).

### Statistical analysis

Statistical analysis was performed using GraphPad Prism software. Two‐tailed Student's *t* tests were used to calculate statistical significance of the difference in sample means. *P* values of less than 0·05 were considered statistically significant. In all figures, data represent the mean ± the standard error of the mean (SEM). *P* values correlate with symbols as follows: ns = not significant, *P ≤ 0·05, **P ≤ 0·01, ***P ≤ 0·001, ****P ≤ 0·0001.

## RESULTS

### CCR8 is highly expressed by tumour‐infiltrating Treg cells and a subset of Tconv cells within tumours

**FIGURE 1:**
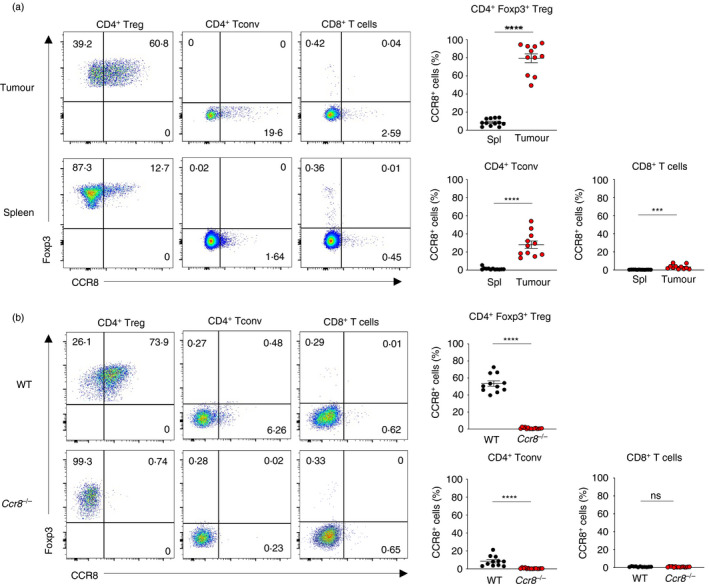
High levels of CCR8 expression discriminate Foxp3^+^ Treg cells within subcutaneously implanted syngeneic MC38 colorectal adenocarcinoma tumours. (a) Representative flow cytometry (left) and replicate measurements (right) of CCR8 expression on indicated CD4^+^ and CD8^+^ Tcell subsets within tumours and spleens of MC38 tumour‐bearing animals at day 21 following tumour implantation. (b) Representative flow cytometry (left) and replicate measurements (right) of CCR8 antibody staining on Treg and CD4^+^ Tconv and CD8^+^T cells within MC38 tumours of WT and *Ccr8* ^−/−^ animals at day 21 following tumour implantation. Data are representative of 2 independently repeated experiments. Bars and error represent mean and SEM. Student's *t* test; ***P < 0·001; ****P < 0·0001; ns, not significant

**FIGURE 2:**
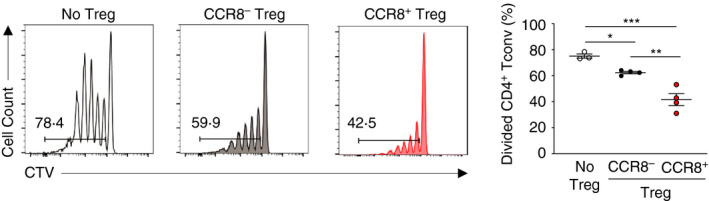
CCR8 marks highly suppressive Foxp3^+^ Treg cells within MC38 colorectal adenocarcinoma tumours. Representative flow cytometry (left) of CTV‐labelled naïve CD4^+^ Tconv cells incubated with no Treg cells, or at a 4:1 ratio with intratumoral CCR8^−^ Treg cells or CCR8^+^ Treg cells from *Foxp3* ^EGFP‐DTR^ mice after 4 days incubation, and replicate measurements of Tconv cell division (right). Data are representative of 2 independently repeated experiments. Bars and error represent mean and SEM. ordinary one‐way ANOVA; *P < 0·05;**P < 0·01 ***P < 0·001

To examine the expression of CCR8 on tumour‐infiltrating T cells, we subcutaneously implanted syngeneic MC38 colorectal adenocarcinoma cells into wild‐type (WT) C57BL/6 animals. Flow cytometry analysis of tumours revealed high levels of CCR8 expression in a substantial fraction of Foxp3^+^ CD4^+^ T cells and a smaller fraction of CD4^+^ and CD8^+^ Tconv cells within tumours of tumour‐bearing animals, whereas CCR8 expression within corresponding T cell populations in systemic lymphoid tissues was substantially lower (Figure 1a). To test the specificity of this signal and of the antibody used in these experiments, we examined anti‐CCR8 antibody staining on the surface of cells within MC38 tumours implanted in WT and *Ccr8* ^−/−^ animals. Whereas a substantial proportion of Foxp3^+^ Treg cells within tumours of WT animals were positive for anti‐CCR8 antibody staining, this signal was abolished upon cells infiltrating tumours of *Ccr8* ^−/−^ animals, confirming both the target and specificity of the antibody used (Figure 1b). CCR8^+^ Treg cells have been previously described as having enhanced suppressive potential compared to their negative counterparts [34, 39]. In order to test in our model whether CCR8 expression marks Treg cells with enhanced suppressive function, we sorted CCR8^−^ and CCR8^+^ intratumoral Treg cells by FACS and tested their capacity to suppress proliferation of autologous naïve CD4^+^ Tconv cells *in vitro* (Figure 2). Both CCR8^−^ and CCR8^+^ Treg cells were capable of suppressing proliferation at 1:4 Treg cell:Tconv cell ratio. However, CCR8^+^ Treg cells had higher suppressive capacity.

### Loss of CCR8 expression does not affect the growth of subcutaneously implanted syngeneic tumours

**FIGURE 3:**
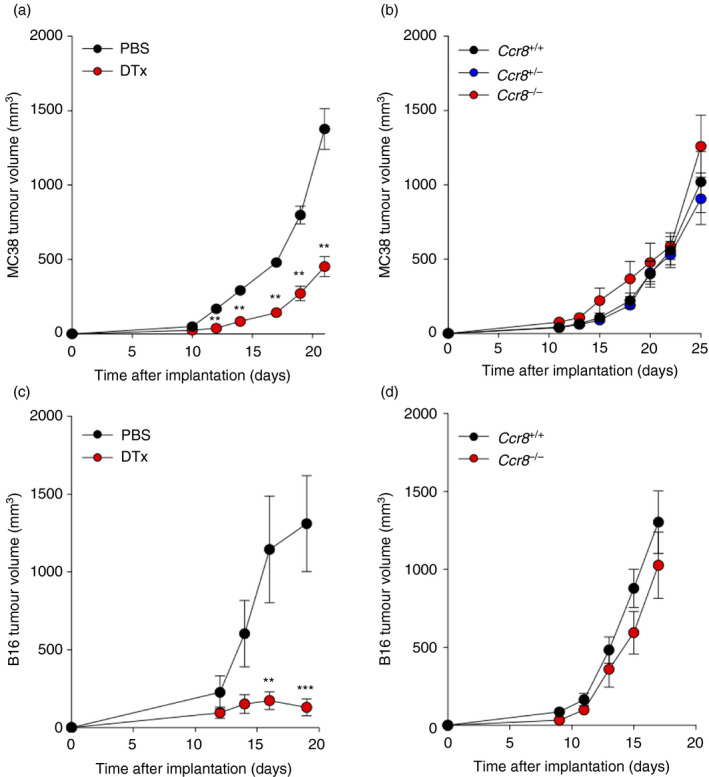
Systemic loss of CCR8 does not affect growth of subcutaneously implanted MC38 or B16‐F10 tumours in contrast to total Treg cell ablation. (a) Volume of heterotopic MC38 colorectal adenocarcinoma tumours at indicated time‐points following implantation into *Foxp3* ^EGFP‐DTR^ animals which were administered with phosphate‐buffered saline (PBS) or diphtheria toxin (DTx) on days 7, 9, 11 and 14. (b) Volume of heterotopic MC38 colorectal adenocarcinoma tumours at indicated time‐points following implantation into animals of the indicated genotypes. (c) Volume of heterotopic B16‐F10 melanoma tumours at indicated time‐points following implantation into *Foxp3* ^EGFP‐DTR^ animals which were administered with PBS or DTx on days 7, 9, 11 and 14. (d) Volume of heterotopic B16‐F10 melanoma tumours at indicated time‐points following implantation into animals of the indicated genotypes. *n* = 5–9 animals per genotype. Data are representative of 2 independently repeated experiments. Bars and error represent mean and SEM. Student's *t* test; **P < 0·01; ***P < 0·001

To test the function of CCR8 in anti‐tumour immunity, we measured the growth of subcutaneously implanted MC38 tumours in littermate WT and *Ccr8* ^−/−^ animals and compared this to the effect of systemic experimental ablation of Treg cells using *Foxp3* ^EGFP‐DTR^ mice, which express human diphtheria toxin receptor (DTR) and enhanced green fluorescent protein (EGFP) under the transcriptional control of the endogenous *Foxp3* gene, enabling selective depletion of Foxp3^+^ Treg cells through administration of diphtheria toxin (DTx) [36]. Whereas systemic ablation of Treg cells resulted in substantially reduced growth of MC38 tumours (Figure 3a), systemic loss of CCR8 expression had no significant effect on tumour growth (Figure 3b). Importantly, we had similar observations using the syngeneic B16‐F10 melanoma tumour model, growth of which was highly sensitive to Treg cell depletion (Figure 3c) but not to germline ablation of *Ccr8* (Figure 3d). These findings suggest that CCR8 function does not have a measurable effect on tumour growth using a syngeneic tumour model highly sensitive to the suppressive function of Treg cells.

### Loss of CCR8 expression does not affect Treg cell accumulation or activation of CD4^+^ or CD8^+^ Tconv cells within tumours

**FIGURE 4:**
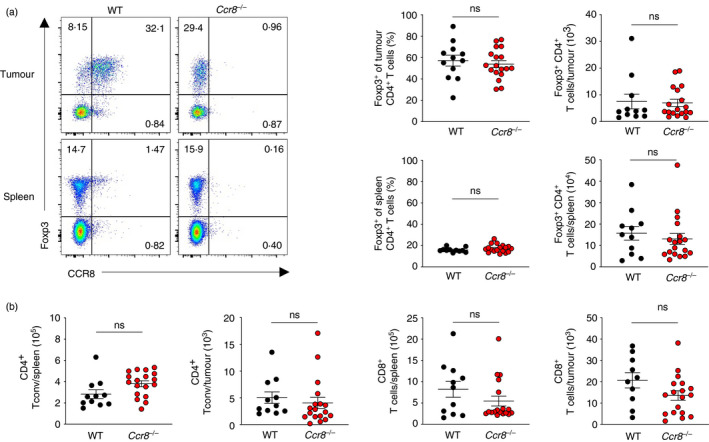
CCR8 expression is dispensable for Foxp3^+^ Treg cell accumulation within tumours. (a) Representative flow cytometry (left) and replicate measurements (right) of Foxp3^+^ Treg cells within spleens and MC38 tumours of WT and *Ccr8* ^−/−^ animals at day 21 following tumour implantation. (b) Representative flow cytometry (left) and replicate measurements (right) of CD4^+^ and CD8^+^ Tconv cells within spleens and MC38 tumours of WT and *Ccr8* ^−/−^ animals at day 21 following tumour implantation. *n* = 11–18 mice per genotype. Data are representative of 4 independently repeated experiments. Bars and error represent mean and SEM. Student's *t* test; ns, not significant

**FIGURE 5:**
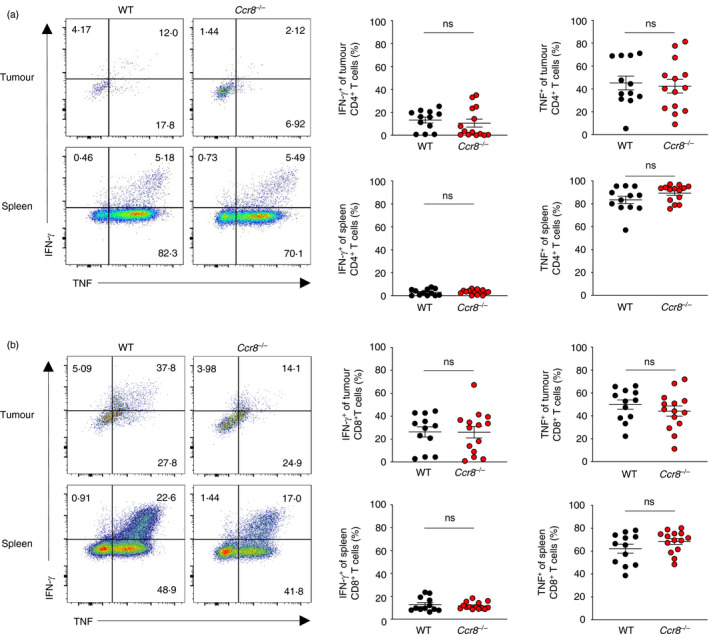
CCR8 expression does not impact suppression of CD4^+^ or CD8^+^ Tconv activation within tumours. (a) Representative flow cytometry (left) and replicate measurements (right) of IFN‐γ and TNF expression as detected by intracellular cytokine staining of CD4^+^ Tconv cells from spleens and MC38 tumours of WT and *Ccr8* ^−/−^ animals at day 21 following tumour implantation. (b) Representative flow cytometry (left) and replicate measurements (right) of IFN‐γ and TNF expression as detected by intracellular cytokine staining of CD8^+^ T cells from spleens and MC38 tumours of WT and *Ccr8*‐KO animals at day 21 following tumour implantation. *n* = 11–18 mice per genotype. Data are representative of 4 independently repeated experiments. Bars and error represent mean and SEM. Student's *t* test; ns, not significant

To formally test the function of CCR8 in Treg cell accumulation within tumours, we examined the frequency and number of Foxp3^+^ Treg cells within MC38 tumours implanted in WT and *Ccr8* ^−/−^ animals (Figure 4a). This analysis revealed that loss of CCR8 function does not affect the frequency or total number of Treg cells within tumours and spleens. No increase was observed in the number of CD4^+^ or CD8^+^ Tconv cells within tumours of *Ccr8* ^−/−^ animals (Figure 4b). Consistently, we did not observe increased production of the type I cytokines IFN‐γ and TNF among CD4^+^ Tconv (Figure 5a) and CD8^+^ (Figure 5b) T cells within tumours or spleens. Collectively, these findings suggest that CCR8 function does not substantially affect anti‐tumour immune responses in the syngeneic MC38 colorectal adenocarcinoma model, despite its sensitivity to Treg cell ablation.

## DISCUSSION

The immunosuppressive function of Treg cells is an important therapeutic target in the immunotherapy of cancer. However, Treg‐targeted therapies should ideally spare the systemic anti‐inflammatory function of Treg cells in other tissues. There is consequently considerable interest in understanding whether Treg cells within tumours possess unique molecular characteristics enabling their selective targeting, either through functional disruption or cellular depletion. Recent studies have identified high levels of CCR8 expression as a distinguishing feature of Treg cells within tumours. It has also been proposed, through experiments where anti‐CCR8 antibodies have been systemically administered, that blockade of CCR8 function impairs the ability of Treg cells to suppress anti‐tumour immunity [34]. Here, we formally tested the contribution of CCR8 to anti‐tumour immunity using genetic loss‐of‐function experiments in mice. We found that CCR8 expression was dispensable both for Treg cell accumulation within tumours and for their immunosuppressive function. CCR8 is also reported to be expressed by Th2 cells, monocytic cells and NK cells. We observed no changes in the frequency of total CD4^+^ Tconv cells in the spleens or tumours of *Ccr8* ^−/−^ mice compared to *Ccr8* ^+/+^ animals but did not in this study examine whether there were differences in the composition of the CD4^+^ Tconv compartment. In addition, the contribution of CCR8 to the function of NK cells and monocytes within tumours was not resolved. Thus, while we observed no overall difference in Treg cell infiltration and tumour immunity in the absence of CCR8, it will be important to examine its functions in greater cellular and molecular resolution in future studies.

We would like to emphasise that our observations are not inconsistent with the recently reported ability of anti‐CCR8 antibodies to reduce tumour growth in syngeneic tumour models in mice, but suggest a re‐interpretation of the mechanism underlying these observations [34]. In particular, while anti‐CCR8 antibodies may have blocking activity, it is possible that the isotypes used also caused some extent of cellular depletion through ADCC. Indeed, mouse fixed chain receptors can cross‐react with antibodies of the rat IgG2b isotype [35]. Whether the anti‐CCR8 antibodies used functioned in part through induction of Treg cell depletion has yet to be formally tested. The hypothesis that therapeutic depletion of CCR8^+^ cells rather than blockade of CCR8 function leads to induction of anti‐tumour immunity is indeed consistent with recently reported findings that administration of anti‐CCR8 nanobodies with blocking function does not augment tumour immunity, but does so when provided the capability for ADCC [40].

Using *Ccr8*‐deficient mice to confirm the specificity of anti‐CCR8 staining, our findings validate prior conclusions that Treg cells infiltrating MC38 colorectal adenocarcinoma tumours express high levels of CCR8 on their cell surface. Thus, depletion of CCR8‐expressing cells remains a potentially important therapeutic approach. Our findings therefore do not lessen the importance of CCR8 as a potential target in therapies aimed at selectively targeting tumour‐associated Treg cells, but suggest that therapeutic depletion of CCR8^+^ Treg cells rather than blockade of CCR8 function is likely to be more efficacious.

## CONFLICT OF INTERESTS

The authors have no competing interests to declare.
